# Bifidobacterial recombinant thymidine kinase-ganciclovir gene therapy system induces FasL and TNFR2 mediated antitumor apoptosis in solid tumors

**DOI:** 10.1186/s12885-016-2608-3

**Published:** 2016-07-27

**Authors:** Changdong Wang, Yongping Ma, Qiongwen Hu, Tingting Xie, Jiayan Wu, Fan Zeng, Fangzhou Song

**Affiliations:** Department of Biochemistry & Molecular Biology, Molecular Medicine & Cancer Research Center, Chongqing Medical University, Yuzhong District, Yi XueYuan Road, No 1, Chongqing, 400016 People’s Republic of China

**Keywords:** Tumor gene therapy, *Bifidobacterium*, Apoptosis, Thymidine kinase, Ganciclovir

## Abstract

**Background:**

Directly targeting therapeutic suicide gene to a solid tumor is a hopeful approach for cancer gene therapy. Treatment of a solid tumor by an effective vector for a suicide gene remains a challenge. Given the lack of effective treatments, we constructed a bifidobacterial recombinant thymidine kinase (BF-rTK) -ganciclovir (GCV) targeting system (BKV) to meet this requirement and to explore antitumor mechanisms.

**Methods:**

*Bifidobacterium* (BF) or BF-rTK was injected intratumorally with or without ganciclovir in a human colo320 intestinal xenograft tumor model. The tumor tissues were analyzed using apoptosis antibody arrays, real time PCR and western blot. The colo320 cell was analyzed by the gene silencing method. Autophagy and necroptosis were also detected in colo320 cell. Meanwhile, three human digestive system xenograft tumor models (colorectal cancer colo320, gastric cancer MKN-45 and liver cancer SSMC-7721) and a breast cancer (MDA-MB-231) model were employed to validate the universality of BF-rTK + GCV in solid tumor gene therapy. The survival rate was evaluated in three human cancer models after the BF-rTK + GCV intratumor treatment. The analysis of inflammatory markers (TNF-α) in tumor indicated that BF-rTK + GCV significantly inhibited TNF-α expression.

**Results:**

The results suggested that BF-rTK + GCV induced tumor apoptosis without autophagy and necroptosis occurrence. The apoptosis was transduced by multiple signaling pathways mediated by FasL and TNFR2 and mainly activated the mitochondrial control of apoptosis *via* Bid and Bim, which was rescued by silencing *Bid* or/and *Bim*. However, BF + GCV only induced apoptosis *via* Fas/FasL signal pathway accompanied with increased P53 expression. We further found that BF-rTK + GCV inhibited the expression of the inflammatory maker of TNF-α. However, BF-rTK + GCV did not result in necroptosis and autophagy.

**Conclusions:**

BF-rTK + GCV induced tumor apoptosis mediated by FasL and TNFR2 through the mitochondrial control of apoptosis *via* Bid and Bim without inducing necroptosis and autophagy. Furthermore, BF-rTK + GCV showed to repress the inflammation of tumor through downregulating TNF-α expression. Survival analysis results of multiple cancer models confirmed that BF-rTK + GCV system has a wide field of application in solid tumor gene therapy.

## Background

Cancer gene therapy approaches include the direct killing of tumor cells by injecting a therapeutic gene into the tumor cell or employing vaccine strategies to deliver an immunomodulatory gene that stimulates the immune system to recognize tumor antigens [1]. *Bifidobacteria* (BF) are an important group of the human intestinal microbiota that exert a number of beneficial probiotic effects on the host, including immunomodulation [2], antibacterial activity [3], bacteriocin production [4], improvement of the intestinal microbial balance [5], and a reduction of inflammation [6]. BF is used in the health care and food industries as a probiotic. BF can target to the hypoxic environment of solid tumors and has been considered to be an alternative strategy in tumor therapy or as a live vaccine [7, 8].

The Herpes Simplex Virus thymidine kinase/ganciclovir (HSV-TK + GCV) system is currently one of the best-studied tumor suicide gene therapy systems [9–11]. When expressed in tumors, TK converts the non-toxic precursor GCV into GCV- 3-phosphate, a toxic substance that kills tumor cells. Apoptotic signaling is initiated either through extrinsic or intrinsic stimulation, resulting in the activation of caspases [12].

We previously found that bladder tumor growth was significantly reduced in rats treated with BI-TK + GCV after 15 days of treatment [10]. However, the mechanism was unclear. In this research, we constructed a BF-specific plasmid pBEX as an expression vector to express TK [8]. A colorectal cancer model was used to decipher the molecular mechanism of BF-rTK + GCV (bifidobacterial recombination thymidine kinase/ganciclovir) using a human apoptosis antibody array kit in a murine cancer model *in vivo*. Another three human cancer xenograft models (gastric cancer MKN-45, liver cancer SSMC-7721 and breast cancer MDA-MB-231) were also established for survival analysis after BF or BF-rTK + GCV intratumor treatment.

## Methods

### Bacterial strains and growth conditions

*E*scherichia *coli* DH5α was used as the host for molecular cloning; pBEX was constructed by MA *et al.* [8] and used as the expression vector in Bifidobacterium (BF). The *Bifidobacterium infantis* strain (Collection in our laboratory) was cultured in MRS broth (Difco) containing 0.25 % (w/v) L-cysteine. HCl (pH 7.0) at 37 °C under anaerobic conditions. Ampicillin (50 mg/ml) was added to both recombinant BF and *E. coli* strains when required.

### Construction of BF-rTK + GCV suicide gene therapy system

HSV TK gene (accession AB032875) was PCR amplified and sub-cloned into pBEX at the *Bam*H I and *Sal* I sites with an artificial signal peptide. Potential recombinants were first screened by bacterial colony PCR. The potential recombinant plasmid was transformed into competent *B. infantis* cells *via* electroporation, signatured BF-rTK were used as TK producer cells, and verified by DNA sequencing.

An intravenous (i.v.) gene therapy in nude mice indicated that 1.0 × 10^6^ cells/ml of BFTK was the highest concentration with no adverse effects, whereas 1.0 × 10^4^ cells/ml was the lowest effective concentration. At concentrations greater than 1.0 × 10^7^ cells/ml, the i.v. injection resulted in venous embolisms and subsequent death. Based on these results, 2.0 × 10^5^ cells/ml were the dosage of BF-rTK used in this study.

BF or BF-rTK (pBEX-*tk*) cells (0.5 ml, 2.0 × 10^5^ cell/ml) were prepared and mixed with 1.0 ml GCV (5.0 mg/kg) respectively and PBS was added to adjust the final volume to 2.0 ml. The negative control was 1.0 ml PBS mixed with 1.0 ml GCV (5.0 mg/kg). Mixtures were incubated at 37 °C for 1.0 h and further incubated for 10 min at 95 °C to stop the reaction. To identify whether the rTK in BF-rTK cells was secreted expression, the 1.0 ml supernatant of BF-rTK culture was isolated by centrifugation for 10 min at 12,000 rpm and incubated with1.0 ml GCV (5.0 mg/kg) at 37 °C for 1.0 h and incubated for another 10 min at 95 °C. The reactants were centrifuged for 10 min at 12,000 rpm. Both supernatants were analyzed by HPLC with an octadecylsilane chemically bonded silica column. The mobile phase ratio was methanol: H_2_O (5:95) and the UV detection wavelength was 252 nm.

### Experimental animals

Mice (Balb/c-nu) and Balb/c mice were housed at the Laboratory Animal Center of Chongqing Medical University (Chongqing, China). This study was carried out in strict accordance with the recommendations in the Guide for the Care and Use of Laboratory Animals of the National Institutes of Health. The protocol was approved by the Committee of the Ethics of Animal Experiments at the Chongqing Medical University (SYXK2012-0001). All procedures were performed under sodium pentobarbital anesthesia, and the method of euthanasia was cervical dislocation.

### Cells and cell culture

Colo320 cell line was obtained from China Center for Type Culture Collection (CCTCC GDC 042), gastric cancer (MKN-45), liver cancer (SSMC-7721) and breast cancer (MDA-MB-231) were obtained from Committee of Type Culture Collection of Chinese Academy of Sciences (CTCCCAS) and maintained in complete growth medium: RPMI 1640 medium with 2 mM L-glutamine adjusted to contain 1.5 g/L sodium bicarbonate, 4.5 g/L glucose, 10 mM HEPES, and 1.0 mM sodium pyruvate, 90 %; 10 % fetal bovine serum. The cells were cultured in 100-mm culture dishes in a humidified, mixed environment of 37 °C and 5 % CO_2_.

### Establishment of xenograft tumor models and experimental groups

Mouse model of xenograft tumor was established by injecting Colo320 cell (1.0 × 10^8^ cells/ml) subcutaneously. Twenty-four tumor-bearing nude mice (male, 3–4 week, 20 g/mouse) were randomly divided into five groups at 7 weeks post-inoculation: the normal control PBS group (*n* = 3), GCV (*n* = 3), PBS + GCV (*n* = 6), BF + GCV (*n* = 6), and the BF-rTK + GCV group (*n* = 6). Each group was once off directly given PBS, GCV, PBS + GCV, BF + GCV, or BF-rTK + GCV through intratumor injections (BF or BF-rTK was 1.0 × 10^6^ cell/tumor, GCV was 5.0 mg/kg). Three tumors were cut from sacrificed mice in each of the last three groups (PBS + GCV, BF + GCV, or BF-rTK + GCV) 48 h postinjection. From each cut out tumor, 20 % was used for immunochemistry analysis and the other 80 % of the tumors of the three mice were mixed together for protein array analysis (n =3). mRNA samples were extracted from three tumors from the last three groups for real time PCR analysis (*n* = 3). From the PBS and GCV groups, mRNA samples were extracted from three tumors for real time PCR analysis (*n* = 3).

### Apoptosis array analysis

Total protein was extracted and prepared from the colo320 tumor xenograft tissues and treated with PBS + GCV, BF + GCV, and BF-rTK + GCV respectively and the proteins concentration was normalized to 10 mg/ml, following the protocol of RayBiotech human apoptosis antibody array kit (Cat# AAH-APO-1-4). The results were analyzed using the RayBiotech cytokine antibody arrays Tool and the ratio of the significant differential expression was considered to be more than 2.0 or less than 0.5.

### Gene silencing and western blotting analysis

Colo320 cells were treated with commercial synthetic small interference RNA (Bim394, Bid77, Bim394+ Bid77, negative control) for 48 h respectively and then treated with or without BF-rTK + GCV for 48 h (with three replicates). Then the cells were lysed with NP40 buffer (1 % NP-40, 0.15 M NaCl, 50 mM, Tris, pH 8.0) containing protease inhibitors (Sigma). Protein quantitation was performed by BCA protein assay reagent (Pierce, USA). Equal amounts of protein from the different groups were denatured in SDS sample buffer and separated on 8–10 % polyacrylamide-SDS gel based on the protein molecular weight. Proteins were transferred to a polyvinylidene difluoride membrane. The antibodies to Bim (abcam 32158), Bid (abcam 32060), GAPDH (cell signaling technology, 14C10) were used to detect the target proteins, followed by incubation with a secondary antibody conjugated with horseradish peroxidase. The proteins of interest were detected using SuperSignal West Pico Chemiluminescent Substrate kit.

### Immunohistochemistry staining

Immunohistochemistry (IHC) of XIAP (E3 ubiquitin-protein ligase XIAP), FADD (FAS-associated death domain protein), APAF-1 (apoptotic protease-activating factor 1) and cleaved Caspase-3 was conducted on five colo320 tumor xenograft tissues treated by PBS, GCV (resolved in PBS solution), BF, BF + GCV and BF-rTK + GCV, respectively (with three replicates). Retrieved tissues were fixed, decalcified in 10 % formalin and embedded in paraffin 24 h posttreatment. Serial sections of the embedded specimens were stained with hematoxylin and eosin (H & E). The fixed tissues of colo320 intestinal tumor were blocked and incubated with XIAP antibody (ab21278, abcam), FADD antibody (ab52935), APAF-1 antibody (ab32372) and cleaved Caspase-3 antibody (ab52293). After being washed, tissues were incubated with biotin-labeled secondary antibody for 30 min, followed by incubation with streptavidin-HRP conjugate for 20 min at RT. The presence of the expected protein was visualized by DAB staining and examined under a microscope. Stains with control IgG were used as negative controls.

### Immunofluorescence

Immunofluorescence staining analysis of FasL (Fas ligand) expression in mouse colo320 tumor xenograft tissues was performed (with three replicates). The slides were then incubated with primary antibody diluted in PBS containing 1 % BSA for 16 h at 4 °C. The primary antibodies used were as follows: anti-FasL antibody (ab68338, 1:500). After washing three times in PBS, Alexa Fluor 55 5-conjugated anti-rabbit IgG (Invitrogen, Grand Island, NY) was added in PBS with 1 % BSA for 1 h. In the final washes, 6-diamidino-2-phenylindole (DAPI) (Sigma) was added and used as a counterstain for nuclei. Fluorescence images were acquired using a Zeiss Axioimager microscope.

### RNA isolation and quantitative RT-PCR

The Caspase-3 downstream effectors (Rock-1 (Rho-associated protein kinase 1), Cad and Acinus (apoptotic chromatin condensation inducer in the nucleus)) were not contained in the apoptosis antibody array. In order to make up for the above mentioned missing in the apoptosis antibody array, total RNA was extracted from three colo320 tumor xenograft tissues from each group treated by PBS + GCV, BF + GCV and BF-rTK + GCV respectively, using TRIzol reagent (Invitrogen). The total RNA was applied to an RNase column (Qiagen, Venlo, Netherlands) for further purification and treated with DNase following the manufacturer’s protocol. cDNA was synthesized from 1 μg of total RNA using the SuperScript III reverse transcriptase kit (Invitrogen) resulting in a final volume of 20 μl. Primers were designed with the IDT SCI primer design tool (Integrated DNA Technologies, San Diego, California). Quantitative real time PCR (qRT-PCR) experiments were performed with Bio-Rad MJ MiniOption Real Time PCR System in triplicate and the data analysis was carried out by the CFX manager software version 1.5. The PCR data were normalized to GAPDH expression. The sequences of each primer pair were listed in Table 1.

**Table 1:** Primers and SiRNA sequences used in this study

Name	Sequence
GAPDH sense antisense Acinus sense Acinus antisense CAD sense CAD antisense ROCK-1 sense ROCK-1 antisense TK sense TK antisense *Bim394 sense* *Bim394 antisence* *Bid77 sense* *Bid77 antisense* ^a^ *NC sense* ^a^ *NC antisence*	5´ ACCACAGTCCATGCCATCAC 3´ 5´ TCCACCACCCTGTTGCTGTA 3´ 5´ AGGTGAGGAGAAGGAGGAAGT 3´ 5´ TCTACTGACACCTGGGGAGG 3´ 5´ CAGCCTCTATGCCAGTCTCG 3´ 5´ CTAGCTGCTCCAGGATGCTC 3´ 5´ GAATGTGACTGGTGGTCGGT 3´ 5´ CTGGTGCTACAGTGTCTCGG 3´ 5´CGCATGGATCCCATGGCTTCGTACCCCTGC 3´ 5´ ACGCGTCGACTCAGTTAGCCTCCCCCATC 3´ *5´ GGUCAUUGGUGAUUAAAUATT 3´* *5´ UAUUUAAUCACCAAUGACCTT 3´* *5´ GGGAUGAGUGCAUCACAAATT 3´* *5´ UUUGUGAUGCACUCAUCCCTT 3´* *5´ UUCUCCGAACGUGUCACGUTT 3´* *5´ ACGUGACACGUUCGGAGAATT 3´*

^a^ *NC* negative control

The italic primers are SiRNA sequences

### Survival rate analysis of the other three kinds of tumor cell lines of nude mouse models in BF-rTK + GCV intratumor treatment

The other three tumor cell lines included gastric cancer (MKN-45), liver cancer (SSMC-7721) and breast cancer (MDA-MB-231). The nude mouse models of xenograft tumor (diameter ≥3.5 mm) were established by injecting the three different kinds of cancer cells (1.0 × 10^8^ cells/ml) subcutaneously. Each positive group contained six nude mice (male, 3–4 week, 20 g/mouse) and when the xenograft tumor diameter was greater than 3.5 mm, BF-rTK (1.0 × 10^6^ cell/mouse) was intratumorally given twice in 5 days. GCV (5.0 mg/kg, *n* = 6) was given via intramuscular injections every day during the five days. Each negative control group of six nude mice bearing the xenograft tumor were raised without any injections (Ctrl, *n* = 6). After the second BF-rTK injection (5 d), all mice were raised without any treatment. The surviving mice were counted every day. The data at the 1 d, 5 d, 17 d, 19 d, 21 d, 24 d, 27 d, 30 d, 35 d and 37 d were used to analyze survival rate. The significant difference was measured by p value.

### Analysis of inflammatory marker in tumor tissue treatment by BF-rTK + GCV

IHC of TNF-α (tumor Necrosis Factor 2 A) was performed on five colo320 tumor xenograft tissues treated by PBS, GCV (resolved in PBS solution), BF, BF + GCV and BF-rTK + GCV, respectively (with three replicates). The following process was the same as the IHC assay of the apoptosis relative markers described previously. The presence of the TNF-α was visualized by DAB staining and examined under a microscope. Stains with control IgG were used as negative controls.

### Effect of BF-rTK + GCV on necroptosis and autophagy protein expression

Necroptosis and autophagy relative protein markers including RIP-1 (Zinc metalloprotease Rip1), ATG5 (autophagy protein 5) and Beclin-1 were analyzed by western blot in colo320 intestinal tumor cell treated with BF + GCV or BF-rTK + GCV. The antibodies of RIP-1 (BA0346-2) and Beclin-1 (BA3123-2) were purchased from Boster (Wuhan, China) and the antibodies of ATG5 (10181-2-AP) were purchased from Proteintech (Wuhan, China).

### Statistical analysis

Statistical analysis was performed using SPSS-17.0 software. Data were analyzed using one-way analysis of variance and Tukey’s HSD test was applied as a post hoc test if statistical significance was determined. Statistical significance for the two groups was assessed using Student’s t-test. The probability level at which differences were considered significant was *p* < 0.05.

## Results

### BF-rTK phosphorylates GCV

**Fig. 1:**
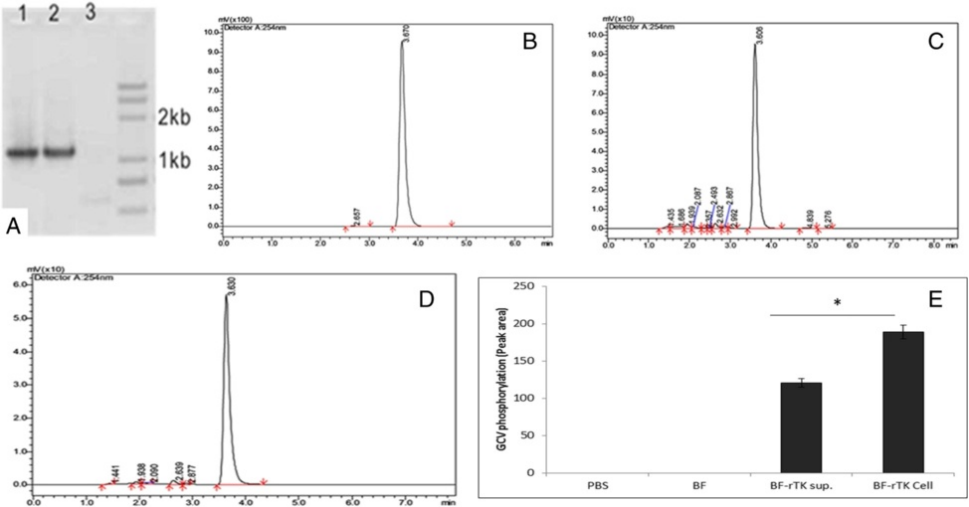
Construction and verification of thymidine kinase expression system and GCV phosphorylated by rTK in *Bifidobacterium infantis*. HPLC detected the concentration of GCV treated with PBS, BF and BF-rTK for 1 h at 37 °C respectively. The supernatants were analyzed by HPLC with octadecylsilane chemically bonded silica column. The mobile phase was methanol + H_2_O (5 + 95) and UV detection wavelength was 252 nm. **a** PCR detection of rTK gene in BF-rTK (lane 1 & 2) and BF (lane 3). **b** PBS + GCV. **c** BF-rTK + GCV. **d** BF + GCV. **e** The BF cell lysis and BF culture supernatant were analyzed by HPLC after incubation with GCV. The BF-rTK supernatant assay suggesting that rTK expressed in BF-rTK cells can be secreted into supernatant (*P < 0.05 statistically significant when comparing treated versus control)

To evaluate the activity of recombinant TK (rTK) expressed in Bifidobacterium (BF), GCV was treated with BF-rTK recombinant and the result showed that 39 % of GCV was phosphorylated by rTK after co-culture for 1 h at 37 °C, and measured using HPLC (Fig. 1b, c, d).

To test that functional rTK was secreted from the recombinant BF cells, the GCV was treated with the supernatant of BF-rTK culture and the result showed that GCV was phosphorylated obviously. The result indicated that the rTK could be secreted by BF (Fig. 1e). However, the level in BF supernatant was less than 43 % (*p* < 0.05).

### BF induces apoptosis *via* Fas/FasL signal pathway and increases Caspase-3, −8 and P53 protein expression levels

In order to evaluate the antitumor activity of bifidobacterium as a gene transfer vehicle as fully as possible, we quantitatively analyzed the colo320 xenograft tumor tissues. This was done by intratumor administration of BF + GCV instead of GCV (in PBS solution) using a RayBiotech human apoptosis antibody array kit containing 43 human apoptotic factors. The results showed that 14 differential proteins expression was upregulated and therefor doubled unlike the expression in those subjected to PBS + GCV (Table 2). Specifically, the expression of Fas (tumor necrosis factor receptor superfamily member 6, TNFRSF6) and FasL was increased more than 2-fold and the changed downstream proteins of Fas/FasL were divided into two groups: four anti-apoptosis proteins (Bcl-2 (apoptosis regulator Bcl-2), Bcl-w, IGF-1 (insulin-like growth factor I), IGF-2) and eight pro-apoptosis proteins (Bad (Bcl2-associated agonist of cell death), Bax (apoptosis regulator BAX), Bim (Bcl-2-like protein 11), Caspase-3,-8, HtrA2 (serine protease HTRA2, mitochondrial), *etc.*; Table 2). The anti-apoptosis proteins were increased more than 5.0-fold. The other eight pro-apoptosis proteins increased from 2.22-fold (FasL) to 8.87-fold (Caspase-8) after BF treatment (Table 2). The ratio of Bax/Bcl-2 was 0.60 in BF treatment. Another characteristic change was a 2.40-fold increase in p53. The total ratio of pro-apoptosis to anti-apoptosis proteins was 2.02-fold. IGF-1 and IGF-2 (insulin-like growth factor) increased more than 5-fold, however, their inhibitor, IGFBPs (IGF binding protein), showed no significant variation in BF + GCV intratumor-treated animals. The downstream effecter, Caspase-3, increased more than 6.56-fold. However, the typical mitochondrial control signal molecule, Cytochrome C (Cyto C), was not significantly changed compared with the PBS + GCV treatment. GCV could not be phosphorylated by *Bifidobacterium*, the variation of Fas/FasL and the downstream proteins were results of the growth of BF in the tumor. Therefore, the results suggested that bifidobacterium itself (not GCV) induced cancer cell apoptosis *via* Fas/FasL signaling pathway without mitochondrial alteration and upregulated P53 expression.

**Table 2:** Apoptosis associated proteins change in group BF + GCV

Gene	PBS + GCV	BF/GCV	Ratio
Bad	335.83	982.79	2.93
Bax	516.54	1567.29^a^	3.03
Bim	192.61	1488.21	7.73
**Bcl-2**	**192.61**	**968.65** ^a^	**5.03**
**Bcl-w**	**192.61**	**1342.08**	**6.97**
**IGF-1**	**192.61**	**984.35**	**5.11**
**IGF-2**	**192.61**	**997.97**	**5.18**
Caspase 3	192.61	1262.99	6.56
Caspase 8	192.61	1708.19	8.87
HtrA2	192.61	1314.83	6.83
Fas	538.63	1201.19	2.23
FasL	521.06	1154.57	2.22
P53	489.92	1175.53	2.40
P27	620.96	1405.44	2.26

The boldface letters were anti-apoptosis proteins and the others were apoptosis proteins

^a^The ratio of Bax/Bcl-2 is 0.60

Up-regulation of fourteen differential proteins expression was more than doubled in the BF + GCV group unlike the expression in the PBS + GCV group. To summarize, the anti-apoptosis proteins were increased more than 5.0-fold. The total ratio of pro-apoptosis to anti-apoptosis proteins was 2.02-fold and the ratio of Bax/Bcl-2 was 0.60. The typical mitochondrial control signal molecule, Cytochrome C (Cyto C), was not significantly changed in group BF + GCV. The results suggested that bifidobacterium itself (not GCV) induced cancer cell apoptosis *via* Fas/FasL signaling pathway without mitochondrial alteration and up-regulated P53 expression

### Effect of BF-rTK/GCV on apoptosis pathway protein expression

The different effects of BF-rTK + GCV and BF + GCV on antitumor activity were also evaluated by the RayBiotech human apoptosis antibody array kit. The results showed that 30 differential proteins in the BF-rTK + GCV intratumor-treated tumor tissues were upregulated more than 2-fold compared with the BF + GCV intratumor-treated group (Table 3). Specifically, 23 pro-apoptosis associated proteins were increased from 2.48-fold (tumor necrosis factor-β, TNF-β) to more than 23.05-fold (Hsp70). Five anti-apoptosis proteins (Bcl-2, Bcl-w, Livin (baculoviral IAP repeat-containing protein 7), IGF-1, IGF-2) were markedly increased from 2.18-fold (IGF-1) to 15.45-fold (Bcl-w) as compared to the BF + GCV group. However, two anti-apoptosis proteins were significantly decreased (XIAP, 0.22-fold, Survivin, 0.28-fold). The total of pro-/anti-apoptosis ratio was 5.20-fold and the ratio of Bax/Bcl-2 was 1.06 in BF-rTK + GCV treatment (Table 3). The P53 protein level was not significantly changed in BF-rTK + GCV group. The IGFs (IGF-1, IGF-2) inhibitors, IGFBP3-6 (IGF binding protein), were increased from 7.39-fold to 8.93-fold as compared to the BF + GCV treated tumors and the ratio of IGFBPs/IGFs was 3.42-fold. The results indicated that BF-rTK + GCV induced the increased expression of many pro-apoptosis associated proteins.

**Table 3:** Apoptosis associated proteins change in group BF-rTK + GCV.

Gene	BF/GCV	BF-rTK/GCV	Ratio
Bad	100.03	895.52	8.95
Bax	100.15	1155.50^a^	11.54
Bid	700.90	3450.87	4.92
Bim	100.38	1292.41	12.88
**Bcl-2**	**100.25**	**1093.11** ^a^	**10.90**
**Bcl-w**	**100.5**	**1552.42**	**15.45**
**XIAP**	**463.48**	**100.25**	**0.22**
**Survivin**	**1047.24**	**296.30**	**0.28**
**Livin**	**100.38**	**605.98**	**6.04**
Caspase 3	100.88	1651.20	16.37
Caspase 8	100.57	1256.01	12.49
Cytochrome C	100.35	1004.71	10.01
HtrA2	100.76	1578.36	15.67
c-IAP2	229.05	779.93	3.41
Fas	100.87	789.76	7.83
FasL	100.35	316.53	3.15
TNF-beta	568.88	1408.11	2.48
TRAILR-1	277.23	1306.83	4.71
TRAILR-2	138.21	1055.52	7.64
TRAILR-3	100.35	411.89	4.11
**IGF-1**	**100.32**	**723.87**	**7.22**
**IGF-2**	**100.77**	**219.51**	**2.18**
IGFBP-3	142.73	1173.35	8.22
IGFBP-4	96.06	857.88	8.93
IGFBP-5	100.32	760.31	7.58
IGFBP-6	100.33	741.30	7.39
Hsp60	100.35	2263.01	22.56
Hsp70	100.89	2325.38	23.05
Hsp27	100.38	1009.85	10.06
P21	472.51	1305.01	2.76

The boldface letters were anti-apoptosis proteins and the others were apoptosis proteins

The BF value was independent of Table 2 and they are read from two different films

^a^The ratio of Bax/Bcl-2 is 1.06

Thirty differential proteins were up-regulated more than 2-fold in group BF-rTK + GCV compared with the BF + GCV intratumor-treated group. To summarize, twenty-three pro-apoptosis associated proteins were increased from 2.48-fold (TNF-β) to more than 23.05-fold (Hsp70). The total of pro-/anti-apoptosis ratio was 5.20-fold and the ratio of Bax/Bcl-2 was 1.06 in group BF-rTK + GCV. The results indicated that BF-rTK + GCV induced the increased expression of many pro-apoptosis associated proteins

### Silencing *Bid* or/and *Bim* expression impairs apoptosis caused by BF-rTK + GCV

**Fig. 2:**
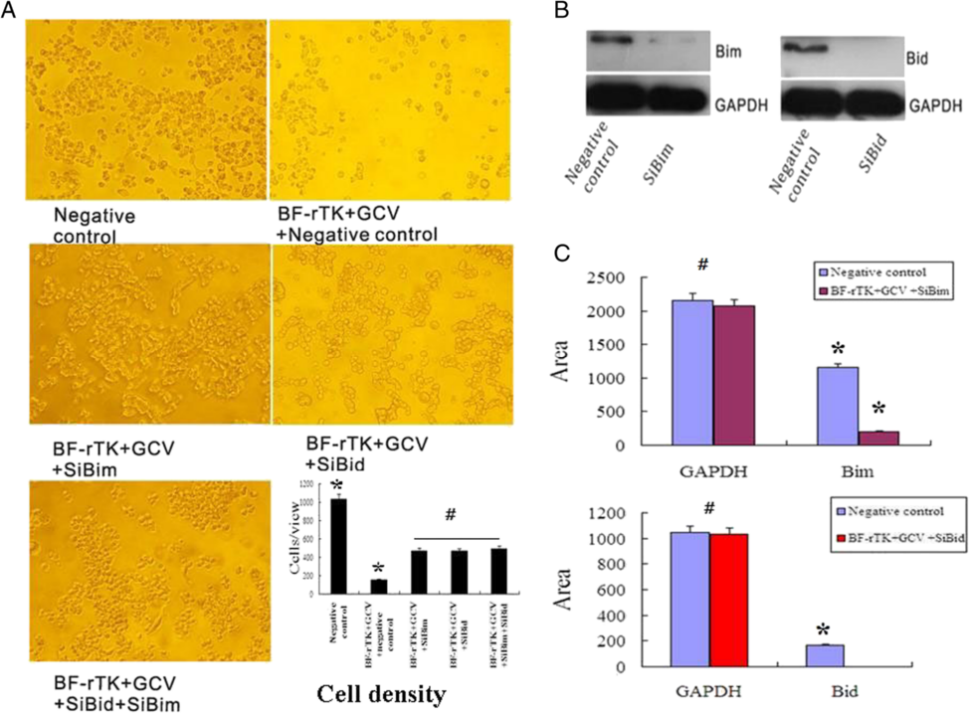
Silencing Bim or/and Bid rescues cancer cells from apoptosis induced by BF-rTK + GCV. **a** Cell imagines and quantitative analysis of the cell density by cell count. (Negative control vs BF-rTK + GCV+ Negative control; BF-rTK + GCV+ Negative control vs BF-rTK + GCV + siBim, BF-rTK + GCV+ Negative control vs BF-rTK + GCV + siBid, BF-rTK + GCV+ Negative control vs BF-rTK + GCV + siBim + siBid). **b** Western blot analysis of Bim and Bid protein expression. Colo320 cells were treated with silencing Bim394, Bid 77 or with a control silencing siRNA for 48 h, and then cells were treated with or without BF-rTK + GCV for 48 h. Cell protein was extracted to detect Bim and Bid expression. Bim or Bid expression was silenced by Bim394 and Bid 77. **c** Quantification of protein levels from immunoblots as in **b**. The protein levels of Bid and Bim were normalized to GAPDH (*P < 0.05 statistically significant when comparing treated versus control; #*P* > 0.05 statistically no significant when comparing treated versus control)

Bid and Bim are two critical signaling proteins located downstream of Fas/FasL (Fas/Fas ligand) and TNF-β/TNFR2 (TNF receptor 2) signal pathway. In order to evaluate their functions in apoptosis, *Bid* or/and *Bi*m were silenced by commercial synthetic siRNA. The cell density was statistically increased in siBim, siBid, or siBim plus siBid treatments together with BF-rTK + GCV unlike in the negative control group which only received BF-rTK + GCV treatment (Fig. 2a). The western blot result showed that Bim (Fig. 2b, left) or Bid (Fig. 2b, right) expression was diminished and/or undetectable in the siRNA Bim394 and Bid77 compared to cells infected with the negative control siRNA. The cell density was dramatically decreased in cells treated with BF-rTK + GCV (Fig. 2a). However, the cell density was significantly increased in siBim, siBid, or siBim plus siBid treatments together with BF-rTK + GCV compared to the group which received BF-rTK + GCV treatment alone (Fig. 2a). Together, these results demonstrated that inhibiting *Bim* or/and *Bid* protein expression could prevent a great amount of cells from apoptosis induced by BF-rTK + GCV.

### Immunohistochemistry (IHC) analysis of active-Caspase-3 and the upstream proteins

**Fig. 3:**
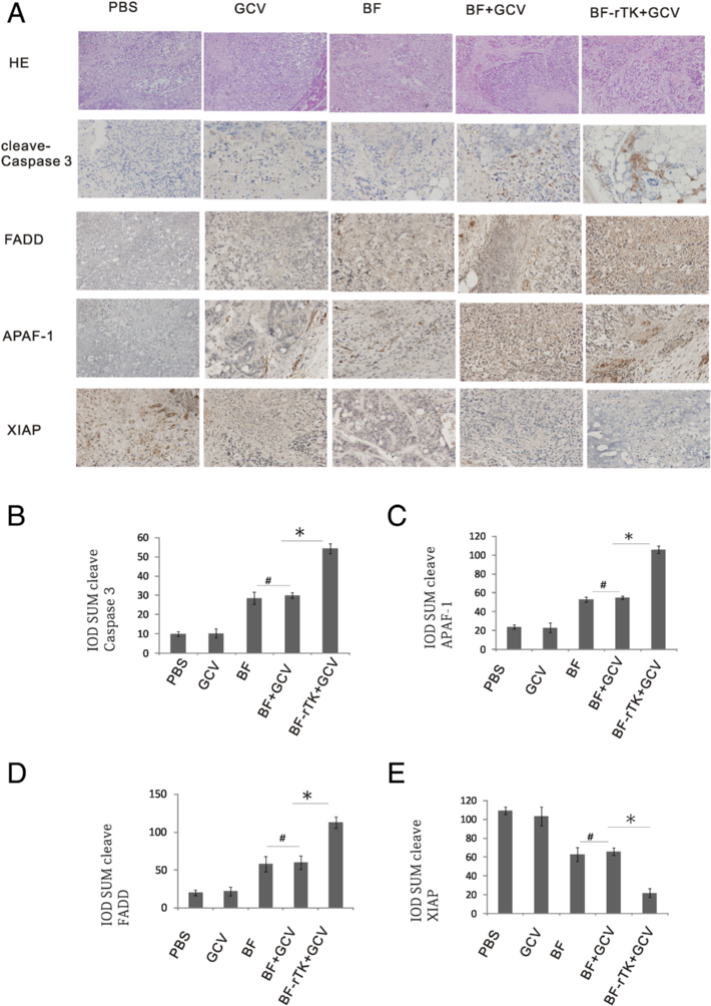
BF-rTK + GCV regulates colo320 tumor xenograft tissues apoptosis, up- regulates FADD, APAF-1 and cleaved caspase-3, and down- regulates XIAP. H&E (hematoxylin-eosin staining) and immunohistochemistry were performed using the specific antibody of cleaved caspase-3, apaf-1, FADD and XIAP antibody and the xenograft tumor tissues treated with PBS, GCV, BF, BF + GCV and BF-rTK + GCV (200×, *n* = 3). **a** Representative histologic sections of H&E staining. The yellow color showed positively stained cells by cleaved caspase-3, apaf-1, FADD and XIAP antibody. **b, c, d** and **e** IOD SUM of positive cells was compared among PBS, GCV, BF, BF + GCV and BF-rTK + GCV mice. Data were given as means and 95 % confidence intervals. Asterisks indicate data that were significantly different from PBS, GCV groups and BF, BF + GCV groups, or both BF, BF + GCV groups and BF-rTK + GCV groups (*P < 0.05 statistically significant when comparing treated versus control)

**Fig. 4:**
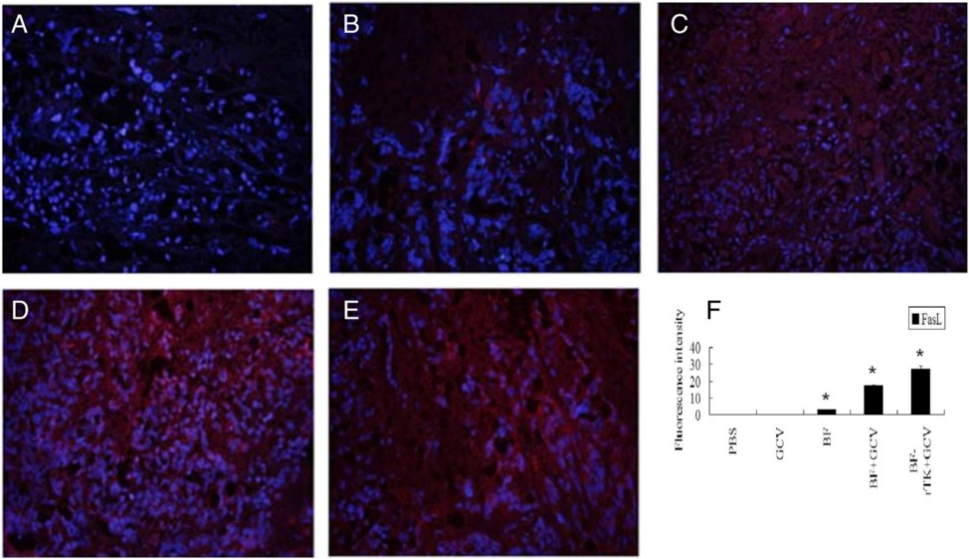
Analysis of FasL in the intestinal colo320 xenograft cancer tissues (*n* = 3) by immunofluorescence. Red is positive for expression of Fasl protein. **a** PBS, **b** GCV, **c** BF, **d** BF + GCV, **e** BF-rTK + GCV (200×). The nuclei were stained with DAPI. **f** Quantitative analysis of the fluorescence intensity from immunofluorescence. (*P < 0.05 statistically significant when comparing treated versus control)

The up-regulated TNF-β, TNFRSFs (tumor necrosis factor receptor superfamily members) and Cytochrom C (Cyto C) in apoptosis antibody array implied that the cancer cell apoptosis was triggered by death receptors and transduced from a Cyto C/Apaf-1/Caspase-9 to Caspase-3 pathway linked to mitochondria. To confirm the hypothesis, several key proteins were analyzed by IHC. The results showed that the cleaved-Caspase-3 (active molecular) up-regulated expression significantly. Meanwhile, the upstream protein, APAF-1, was also upregulated which was crucial for Caspase-3 activation. The FADD was upregulated significantly which was essential to Caspase-8 activation and then transduced signals to mitochondrion and/or Caspase-3. In addition, IHC assay also confirmed that XIAP (Caspase-3 inhibitor) expression decreased in BF-rTK + GCV treatment recipient tissues (Fig. 3a and e). FasL is a stimulator that activates FADD through Fas. FasL immunostaining also revealed that FasL expression was increased significantly in colo320 tumor xenograft tissue intratumorally treated with BF-rTK + GCV (Fig. 4e and f). The results suggest that BF-rTK + GCV triggered many TNF superfamily receptor mediated signal transduction pathways (e.g. Fas, TNFR2 and TNFRSFs (DR4 (death receptor 4, TNFRSF10A), DR5 (TNFRSF10B)) and the signals were transduced through mitochondrial associated caspase-3 pathway.

### Gene transcription of Caspase-3 downstream effectors is significantly up-regulated by BF-rTK/GCV in colo320 intestinal tumor

**Fig. 5:**
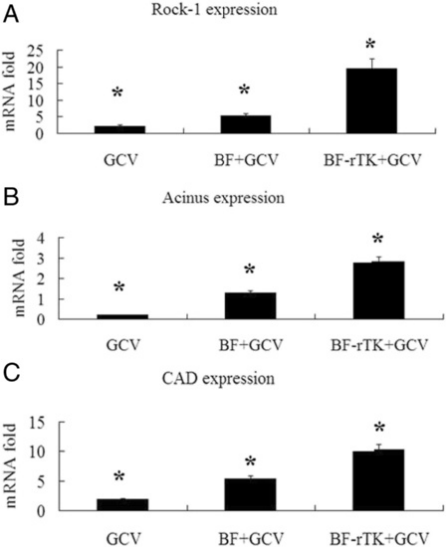
Caspase-3 downstream apoptosis-related genes are up-regulated in BF-rTK + GCV induced apoptosis. **a**–**c** Colo320 intestinal xenograft tumor tissues treated by GCV, BF + GCV and BF-rTK + GCV were collected and RNA was extracted from tumor tissues (*n* = 3). Gene transcription of *Rock-1* (**a**), *Acinus* (**b**) and *Cad* (**c**) were analyzed by qRT-PCR using specific primers. All values were normalized to GAPDH as an internal control and were expressed relative to tumors treated with GCV in each case (*P < 0.05 statistically significant when comparing treated versus control)

Caspase-3 played a crucial role in the TNF superfamily receptor induced apoptosis signaling pathway. The active Caspase-3 induced several effectors activity through three different pathways and induced apoptosis. In order to evaluate the level and type of Caspase-3 downstream pathway activated by BF-rTK + GCV, three Caspase-3 effectors genes (*Rock-1, Cad and Acinus*) were detected using qRT-PCR. The results showed that BF-rTK + GCV triggered *Rock-1*, *Cad* and *Acinus* transcription to increase significantly more than that of the *in vivo* BF + GCV treatment group (3 ~ 21-folds; Fig. 5). The Rock-1 induced cell shrinkage and membrane blebbing, CAD induced DNA fragmentation and Acinus induced chromatin condensation and finally resulted in cell apoptosis. Therefore, the data suggested that Caspase-3 is a key connecting link between the preceding and the following of BF-rTK + GCV induced apoptotic signaling pathway.

### BF-rTK + GCV induces cell apoptosis through TNFR2 signaling in *vitro*

**Fig. 6:**
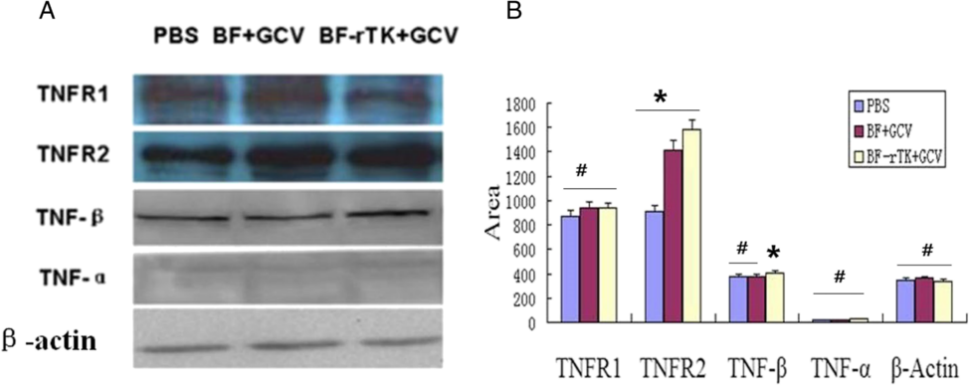
Analysis of TNF and TNFR expressions in BF-rTK + GCV treatment of colo320 intestinal tumor. **a** The protein samples were extracted from colo320 cells treated by PBS, BF + GCV, and BF-rTK + GCV, respectively. The TNF-α, TNF-β, TNFR1 and TNFR2 levels were analyzed with western blot. The gels were run under the same experimental conditions. **b** Quantitative analysis of TNF-α, TNF-β, TNFR1 and TNFR2 (*P < 0.05 statistically significant when comparing treated versus control; #*P* > 0.05 statistically no significant when comparing treated versus control)

To elucidate the type of TNFs and TNFRs interaction in the tumor cell apoptosis induced by BF-rTK + GCV, TNF-α, TNF-β, TNFR1 and TNFR2 were analyzed by western blot in colo320 intestinal tumor cell. The novelty found in this work was that BF-rTK + GCV triggered TNF-β (not TNF-α) to induce cancer cell apoptosis *in vitro* TNFR2 (not TNFR1) (Fig. 6).

**Fig. 7:**
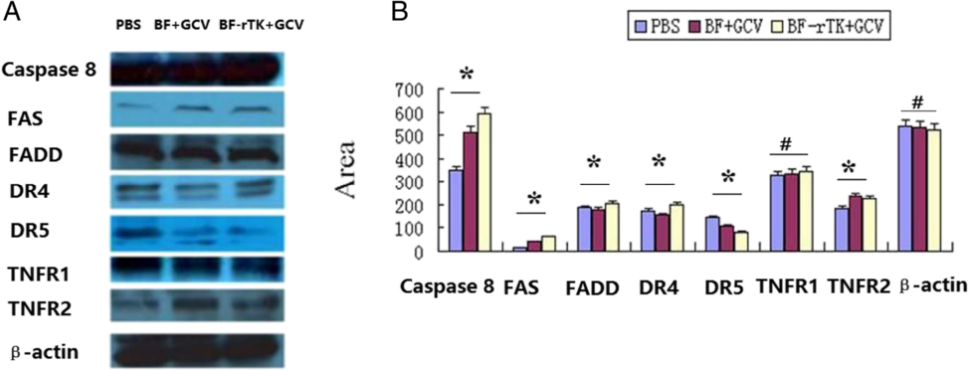
Analysis of apoptosis associated proteins expression in different kinds of cancer cell lines treated by BF-rTK + GCV.** a** The gastric cancer cell MKN-45 proteins were extracted and some typical apoptosis associated proteins (FAS, FADD, DR4, DR5, TNFR1 and TNFR2) were analyzed by western blot with specific antibodies. **b** Quantitative analysis of FAS, FADD, DR4, DR5, TNFR1 and TNFR2 (*P < 0.05 statistically significant when comparing treated versus control; #*P* > 0.05 statistically no significant when comparing treated versus control)

To evaluate the universality of BF-rTK + GCV induced apoptosis *via* TNFR2 mediated signaling pathway, gastric cancer cell (MKN-45) was employed as another model. The apoptosis related proteins, namely, FAS, FADD, active-Caspase-8, TNFR1, TNFR2, DR4 and DR5 were tested with western blot. These results confirmed that BF-rTK + GCV universally induced solid tumor cell apoptosis *via* TNFR2 mediated signaling pathway (Fig. 7).

### BF-rTK + GCV prevented death of a wide variety of solid tumor mice models

**Fig. 8:**
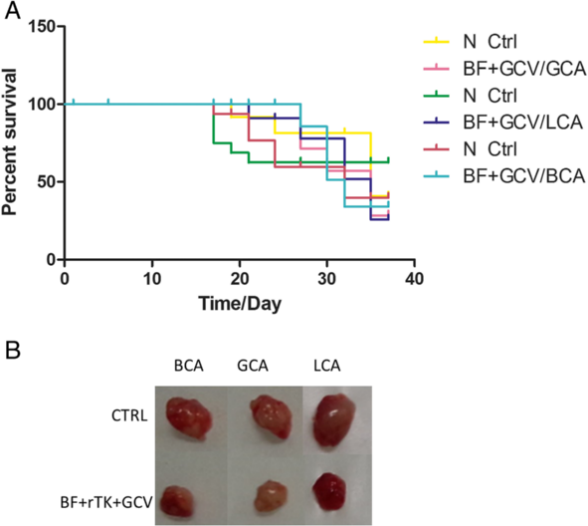
Survival rate analysis of a wide variety of solid tumor mice models in BF-rTK + GCV treatment. **a** Three different kinds of human solid tumor models (gastric cancer MKN-45 (GCA), breast cancer MDA-MB-231 (BCA) and liver cancer SSMC-7721 (LCA)) were established by injecting cancer cells (1.0 × 10^8^ cells/ml) subcutaneously. Each positive group contained six nude mice that had BF-rTK + GCV intratumorally injected twice in five days after the xenograft tumor diameter was more than 3.5 mm (BF-rTK + GCV was 1.0 × 10^6^ cell/tumor, *n* = 6) and GCV (5.0 mg/kg) was given as an intramuscular injection every day during the five days. Each negative control group of six nude mice bearing xenograft tumors were raised without any injections (Ctrl, *n* = 6). After the second BF-rTK + GCV injection for positive groups, all mice were raised without any treatment. The mice that survived were counted every day. The data at the 1 d, 5 d, 17 d, 19 d, 21 d, 24 d, 27 d, 30 d, 35 d and 37 d postinjection were used to analyze survival rate (*P < 0.05). **b** On 32 d postinjection, the tumors were isolated from three different groups and the tumor size was compared between the control and treatment groups

In order to evaluate the universality of BF-rTK + GCV antitumor activity, the survival rate after two intratumor BF-rTK + GCV injections of three different kinds of human solid tumor models (gastric cancer, liver cancer and breast cancer) were analyzed. The results showed that BF-rTK + GCV prevented more than 83 % of tumor bearing mice from death of liver cancer after the mere administration of two doses of intratumor injections. The protection rates were 50 % in the gastric cancer group and breast cancer group during a period of 30 days (Fig. 8a). The tumor growth was inhibited and the treated tumors were smaller on day 32 after treatment (Fig. 8b). There was significant difference between the groups of BF-rTK + GCV treatment and their controls in each of cancer models (*p* < 0.05). However, the statistical difference of BF-rTK + GCV treatment effects between the three groups was no significant (*P* > 0.05). The results suggested that BF-rTK + GCV effectively prevented mice from death in multiple human solid tumor models.

### BF-rTK + GCV inhibits inflammatory marker, TNF-α, expression

**Fig. 9:**
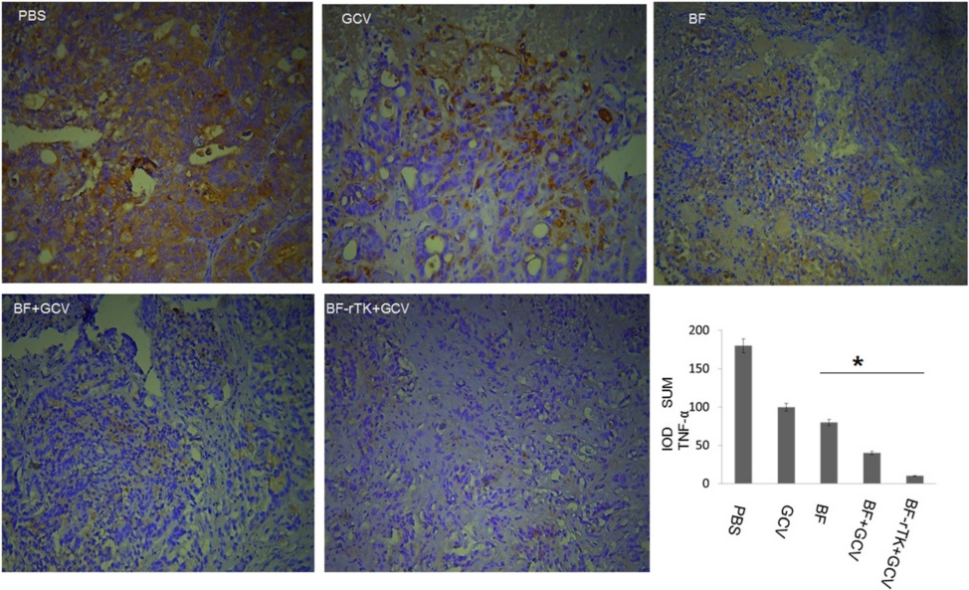
BF-rTK inhibits intestinal cancer tissues TNF-α expression. Immunohistochemistry using the specific antibody of TNF**-**α antibody and the xenograft tumor tissues treated by PBS, GCV, BF, BF + GCV and BF-rTK + GCV (200×, *n* = 3). The yellow color shows positively stained cells by TNF antibody. IOD SUM of positive cells was compared among PBS, GCV, BF, BF + GCV and BF-rTK + GCV mice. Data are given as means and 95 % confidence intervals. Asterisks indicate data that are significantly different from PBS, GCV groups and BF, BF + GCV groups, or both BF, BF + GCV groups and BF-rTK + GCV groups (*p < 0.05 statistically significant when comparing treated versus control)

The IHC of TNF-α result showed that BF-rTK + GCV administration (i.v) significantly down-regulated TNF-α expression (Fig. 9). The result suggested that BF-rTK + GCV administration (i.v) inhibits the expression of the major tumor inflammatory marker, TNF-α, in tumor microenvironment. Correspondingly, the BF-rTK + GCV treatment did not increase the expression of TNFR1 (TNF-α receptor type 1) both in colo320 intestinal tumor cell (Fig. 6) and in gastric cancer cell (MKN-45) (Fig. 7). Therefore, the feature of inflammatory inhibition might be taken advantage of for BF-rTK + GCV cancer treatment.

### Effects of BF-rTK + GCV on necroptosis and autophagy associated protein expression

**Fig. 10:**
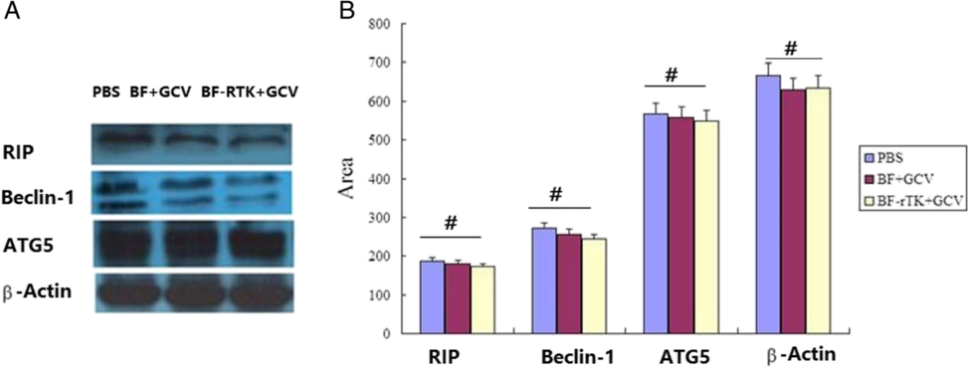
Western blot analysis of necroptosis and autophagy associated proteins expression in BF-rTK + GCV treatment intestinal tumor of colo320. **a** Colo320 intestinal tumor cells were treated by PBS, BF + GCV or BF-rTK + GCV, respectively. The necroptosis and autophagy associated proteins (RIP-1, ATG5, Beclin-1) were detected by specific antibodies from colo320 cell 48 h post-treatment. The cropped gels were run under the same experimental conditions. **b** Quantitative analysis of RIP-1, ATG5 and Beclin-1 (#*P* > 0.05 statistically no significant when comparing treated versus control)

Besides apoptosis, necroptosis and autophagy are two basic cell death pathways [13, 14]. In order to elucidate the effects of BF-rTK/GCV on necroptosis or/and autophagy in cancer cells, the typical molecular marker proteins of necroptosis and autophagy were analyzed with western blot. The results showed that RIP-1 protein expression was slightly down-regulated in colo320 cell treated by BF-rTK + GCV. RIP-1 is a critical mediator of necroptosis. The result suggested that BF-rTK + GCV treatment had no effect on necroptosis (Fig. 9, *P* > 0.05). We further explored whether BF-rTK + GCV can promote or decrease the autophagy related proteins (ATG5, Beclin-1) expression. Similarly, the western blot results also showed no significant change. Therefore, BF-rTK + GCV treatment also had no effect on autophagy (Fig. 10).

Taken together, the first significance of the findings was that BF-rTK + GCV induced tumor apoptosis through multiple signaling pathways mediated by two Fas/FasL and TNF-β/TNFR2 and mainly activated the mitochondrial control of apoptosis *via* Bid and Bim. Caspase-3 played a crucial role as a hub and transduced apoptosis signals through three different pathways *via* Rock-1, CAD and Acinus. The second significance of the findings was that the results of multiple cancer lines analysis confirmed that BF-rTK + GCV system treatment prevented tumor bearing mice from death in a wide variety of solid tumors and the mechanism of apoptosis had universality. The third significance of the findings was that BF-rTK + GCV system treatment had no effect on tumor necroptosis or autophagy.

## Discussion

Conventional suicide gene therapy vectors used in cancer cases are typically based on herpes simplex virus or adenovirus [15, 16]. There are HSV-TK + GCV-mediated gene therapy systems and adenovirus-mediated gene therapy systems and lentivirus TK + GCV gene therapy for lung cancer treatment [9, 17–19]. It is clear that GCV is phosphorylated by the HSV1-TK to GCV monophosphate, and further to GCV di- and triphosphate and incorporated into proliferating tumor cell DNA, which causes DNA chain termination and induces tumor cell apoptosis [17]. The major obstacle for wide clinical application of this approach is the insufficient amounts of the suicide gene delivered into the target tumor tissue by virus-based vectors [17]. For example, the multiplicity of infection (MOI) of adenovirus-Rous sarcoma virus-thymidine kinase is no less than 66 in MDAH-2774 ovarian cancer cells after acyclovir treatment [20].

Bifidobacterium (BF) is a non-pathogenic, non-toxic, and strictly anaerobic gram-positive bacterium and can target the hypoxic environment of solid tumors for its anerotaxis [7, 10, 21]. In this research, we revealed the differences in mechanisms of BF + GCV and BF-rTK + GCV systems in inducing colo320 cell apoptosis in detail. Compared with virus-mediated vectors, the superiority of BF-rTK recombinant is that it does not invade the tumor cell and the rTK can be secreted outside of BF and thereby phosphorylates GCV. The phosphorylated GCV diffuses in the tumor tissue and functions its antitumor activity and the process remains independent of tumor cellular bio-systems. That means, BF-rTK does not target a single cancer cell, but the solid tumor as a whole. However, recombinant viruses (e.g. adenovirus-Rous sarcoma virus-thymidine kinase) have to infect and kill single cancer cells one by one. The bacteria engulfing is not necessary for BF-rTK + GCV system. The BF-rTK recombinant can be quickly reproduced outside cancer cells independently. Therefore, BF can deliver sufficient suicide genes into the target tumor tissue without MOI limitation.

Death receptors (DRs) are the members of TNF receptor superfamily including Fas/FasL, TNFRSF (DR4 (TNFRSF10A), DR5 (TNFRSF10B)) and TNF receptor (TNFR1, 2) [22, 23]. TNF-β, lymphotoxin α, is generally described as an inflammatory and immune response factor and is signaled via TNFR1 and TNFR2. TNF-β is involved in the processes of inducing cell apoptosis when it is signaled by TNFR1. It then subjects a wide range of tumor cells to cytotoxicity [24]. However, there are few reports about TNF-β inducing cancer cell apoptosis *via* TNFR2 to date.

Compared to BF + GCV intratumor treatment, BF-rTK + GCV treatment increased four IGFBPs expression (Table 3). IGFBPs down regulate the activity of IGFs [18], and promote apoptosis by modulating the expression of apoptosis-specific genes such as *Bcl-2* and *Bax* [24–26]. IGFBP-6 has a high affinity for binding IGF-2 and is able to inhibit the growth of various cancer cells and activated apoptosis pathways as an IGF-antagonist [27–29]. The Bax/Bcl-2 ratio in BF-rTK + GCV was increased 1.7-fold compared to BF + GCV treatment (Tables 2 and 3), which lead to the activation of the caspase cascade. However, IGFs promote a shift in the expression of the Bcl-2 family and prevent glucose-induced Cyto C release, which is directionally blocked activation of the terminal apoptosis program and exhibits a decrease in the Bax/Bcl-2 ratio [30]. That could explain why no detectable Cyto C was found in the BF + GCV group. Therefore, the pro-apoptosis proteins overwhelmed the anti-apoptosis proteins and the final results were tilted the balance toward apoptosis (Tables 2 and 3).

XIAP, c-IAP2, Livin and Survivin belong to the inhibitor of apoptosis family (IAP) with typical BIR (baculovirus IAP repeat) domain. IAP directly binds to Caspases as well as neutralization of Smac and further activates downstream anti-apoptotic cascades [30]. However, the inhibitory effect of Livin on Caspase-3 and Caspase-9 is much weaker compared to that of XIAP [31].

Bid and Bim are two important upstream target proteins up-regulated by Fas/FasL signaling and TNFR signaling in the mitochondrial control of apoptosis. *Bid* and/or *Bim* SiRNA treatment prevented colo320 intestinal tumor cells from apoptosis induced by BF-rTK + GCV *in vitro* as expected. The results confirmed that Fas/FasL signaling and TNFR signaling are principal pathways in BF-rTK + GCV induced colo320 intestinal tumor apoptosis *in vivo.* The gene silencing results suggested that these changes are causative rather than simply secondary effects of BF-rTK + GCV treatments.

Inflammation was identified as the seventh feature of cancer [32]. Our data showed that BF-rTK + GCV system inhibited both TNF-α and its receptor, TNFR1, expression in tumor tissue, which indicated that BF-rTK + GCV inhibited inflammation induced by TNF-α/TNFR1 pathway. It was a synergistic effect of the tumor therapy. TNF-α is known to play an important role in various aspects of tumor progression. It was reported that TNF-α may promote breast cancer cell migration by inducing activation of the MAPK/ERK signaling pathway [33]. In another study, TNF-α was found to stimulated prostate carcinogenesis in chemically induced mice by activation of the AKT/mTOR and NFkB pathway [34]. Evidence suggested that the anti-inflammatory treatment prior to chemotherapy suppressed the acquisition of chemoresistance of breast cancer patients [35]. Therefore, BF-rTK + GCV anti-inflammation effect was helpful for overcoming the chemoresistance of cancer.

Hopefully, the BF-rTK + GCV system might overcome drug resistance in single-target drug use in tumor therapy for its multiple targets and multiple effects. Our study highlighted the potential of BF-rTK + GCV system for solid tumor therapy.

## Conclusion

BF-rTK + GCV induced tumor apoptosis mediated by FasL and TNFR2 through the mitochondrial control of apoptosis *via* Bid and Bim t does not result in necroptosis and autophagy. BF-rTK + GCV anti-inflammation effect was useful for overcoming the chemoresistance of cancer. Survival analysis results of multiple cancer models confirmed that BF-rTK + GCV system has a wide field of application in solid tumor gene therapy.

## Abbreviations

APAF-1, apoptotic peptidase activating factor 1; BF, *Bifidobacterium infantis*; BF-rTK + GCV, Bifidobacterium recombination thymidine kinase/ganciclovir; DAPI, 6-diamidino-2-phenylindole; FADD, Fas-associated with death domain protein; HTrA2, HtrA serine peptidase 2; IAP, inhibitor of apoptosis protein; IGF, insulin-like growth factor; IGFBP, insulin-like growth factor-binding protein; MOI, multiplicity of infection; NIK, kappaB-inducing kinase; PBS, phosphate-buffered saline; PCR, Polymerase Chain Reaction; PVDF, polyvinylidene difluoride; SDS, sodium dodecyl sulfate; siRNA, small interference-based RNAi; TNF, tumor necrosis factors; TRAF2, tumor necrosis factor receptor associated factor 2; TRAF2, tumor necrosis factor receptor associated factor-2; XIAP, X-linked inhibitor of apoptosis protein
